# PACAP-38 Induces Transcriptomic Changes in Rat Trigeminal Ganglion Cells Related to Neuroinflammation and Altered Mitochondrial Function Presumably via PAC1/VPAC2 Receptor-Independent Mechanism

**DOI:** 10.3390/ijms23042120

**Published:** 2022-02-14

**Authors:** Krisztina Takács-Lovász, József Kun, Timea Aczél, Péter Urbán, Attila Gyenesei, Kata Bölcskei, Éva Szőke, Zsuzsanna Helyes

**Affiliations:** 1 Department of Pharmacology and Pharmacotherapy, Medical School & Szentágothai Research Centre, Molecular Pharmacology Research Group, Centre for Neuroscience, University of Pécs, H-7624 Pécs, Hungary; takacs-lovasz.krisztina@pte.hu (K.T.-L.); aczel.timea@pte.hu (T.A.); bolcskeikata@outlook.com (K.B.); eva.szoke@aok.pte.hu (É.S.); zsuzsanna.helyes@aok.pte.hu (Z.H.); 2 Szentágothai Research Centre, Bioinformatics Research Group, Genomics and Bioinformatics Core Facility, University of Pécs, H-7624 Pécs, Hungary; urban.peter@pte.hu (P.U.); gyenesei.attila@pte.hu (A.G.)

**Keywords:** pituitary adenylated cyclase-activating polypeptide (PACAP), trigeminal ganglion, transcriptomics, intracellular calcium, mitochondrial electron transport chain

## Abstract

Pituitary adenylate cyclase-activating polypeptide (PACAP) is a broadly expressed neuropeptide which has diverse effects in both the peripheral and central nervous systems. While its neuroprotective effects have been shown in a variety of disease models, both animal and human data support the role of PACAP in migraine generation. Both PACAP and its truncated derivative PACAP(6-38) increased calcium influx in rat trigeminal ganglia (TG) primary sensory neurons in most experimental settings. PACAP(6-38), however, has been described as an antagonist for PACAP type I (known as PAC1), and Vasoactive Intestinal Polypeptide Receptor 2 (also known as VPAC2) receptors. Here, we aimed to compare the signaling pathways induced by the two peptides using transcriptomic analysis. Rat trigeminal ganglion cell cultures were incubated with 1 µM PACAP-38 or PACAP(6-38). Six hours later RNA was isolated, next-generation RNA sequencing was performed and transcriptomic changes were analyzed to identify differentially expressed genes. Functional analysis was performed for gene annotation using the Gene Ontology (GO), Kyoto Encyclopedia of Genes and Genomes (KEGG), and Reactome databases. We found 200 common differentially expressed (DE) genes for these two neuropeptides. Both PACAP-38 and PACAP(6-38) treatments caused significant downregulation of NADH: ubiquinone oxidoreductase subunit B6 and upregulation of transient receptor potential cation channel, subfamily M, member 8. The common signaling pathways induced by both peptides indicate that they act on the same target, suggesting that PACAP activates trigeminal primary sensory neurons via a mechanism independent of the identified and cloned PAC1/VPAC2 receptor, either via another target structure or a different splice variant of PAC1/VPAC2 receptors. Identification of the target could help to understand key mechanisms of migraine.

## 1. Introduction

Pituitary adenylate cyclase-activating polypeptide (PACAP), a neuropeptide belonging to the vasoactive intestinal polypeptide (VIP)-secretin family, is broadly expressed throughout the body. It exists in 27- and 38-amino acid-containing forms (PACAP-27 and PACAP-38), the latter one is the predominant form in mammals. PACAP was shown to have diverse actions in both the central nervous system (CNS) and the periphery. Its physiological roles include the regulation of circadian rhythm, energy homeostasis, as well as the activity of the hypothalamic-pituitary-adrenal axis. PACAP also has a major role in neurodevelopment, neuroprotection and regeneration in a variety of CNS injuries and neurodegenerative processes [1,2]. PACAP exerts most of its effects via the specific PAC1 receptor, as well as the VPAC1 and 2 receptors [3,4,5] shared with VIP having comparable affinities. In addition, Mas-related G-protein coupled receptor (Mrgpr) activation by PACAP-38 or its truncated derivatives, PACAP(6-38) or PACAP(6-27), was described on mast cells [6,7]. Mrgpr receptors belong to a diverse family of receptors, many of which are also expressed on sensory neurons [8]. Still, Mrgpr activation by PACAP has not been explored up to date.

Conflicting results were reported for the effects of PACAP on nociception in rodent models. While electrophysiological recordings showed that PACAP had a direct activating and sensitizing effect on nociceptive neurons, both pronociceptive and analgesic effects were reported in pain models, in vivo. Intrathecally administered PACAP induced nocifensive behavior in mice [9,10], but attenuated formalin-induced responses in rats [11,12]. Peripherally administered PACAP reduced hyperalgesia and pain-related behaviors in acute/subacute models of inflammatory and visceral pain [13]. In PACAP gene-deleted mice, the overall effect of PACAP was concluded to be pronociceptive in a variety of chronic pain models by contributing to central nociceptive sensitization [14,15,16,17].

It is of particular relevance that PACAP was implicated in the generation of migraine headaches. PACAP plasma levels were found to be elevated in migraine patients [18] and PACAP infusion could trigger headache in healthy volunteers and also induced a delayed, migraine-like headache in migraine patients [19,20]. Neurogenic inflammation induced by dural sensory nerve stimulation and consequent release of proinflammatory neuropeptides such as calcitonin-gene related peptide (CGRP), tachykinins and PACAP, is an important component of migraine [21] It consists of meningeal vasodilatation, plasma protein extravasation (oedema formation) and activation of inflammatory cells including mast cells [22], which in turn also release inflammatory mediators i.e., cytokines and peptides, such as PACAP [23]. These mediators further trigger the sensory nerve terminals leading to the aggravation of local neurogenic inflammation and pain [24,25].

Both PACAP and its receptors are expressed in the primary and secondary sensory neurons in the trigeminal ganglia (TG) [26,27] and trigeminal nucleus caudalis (TNC), respectively [4,28]. Animal experiments also confirmed that PACAP induced meningeal vasodilation and neuronal activation in the trigeminovascular system [29,30].

The receptorial mechanisms of PACAP in the trigeminovascular system are not understood. Since the headache-inducing effect of PACAP was not shared with VIP, the contribution of VPAC receptors could be discarded [31]. Additional data also demonstrate that PACAP can induce CGRP release independently of VPAC2 and PAC1 receptors, as well [32]. Since various Mrgprs are also expressed on primary sensory neurons [33,34], it can also be suggested that Mrgpr activation might contribute to the pronociceptive effects of PACAP.

Even though PACAP(6-38) can antagonize the effect of PACAP on heterologously expressed PAC1 receptors and various neuronal cell lines, our previous results clearly showed that PACAP(6-38) treatment did not inhibit PACAP-38, but produced identical effects by itself in rat primary sensory neurons. Both PACAP-38 and PACAP(6-38) could inhibit neuropeptide release from sensory nerve terminals of isolated trachea [35] and induce Ca^2+^-influx in primary cultures of trigeminal ganglion cells [36]. 

In order to elucidate the receptorial and signaling mechanisms of PACAP-induced trigeminal ganglia primary sensory neuronal activation related to migraine, here we analyzed and compared the transcriptome changes in cell cultures treated with PACAP-38 or PACAP(6-38).

## 2. Results

We determined the common differentially-expressed (DE) genes identified 6 h after PACAP-38 and PACAP(6-38) treatment. Administration of the two peptides has a high impact related to migraine-like cellular processes. All data are demonstrated in the Supplementary section.

### 2.1. Expression of PAC1, VPAC2 and Mrgpr Receptor Transcripts in TG Cultures

**Figure 1:**
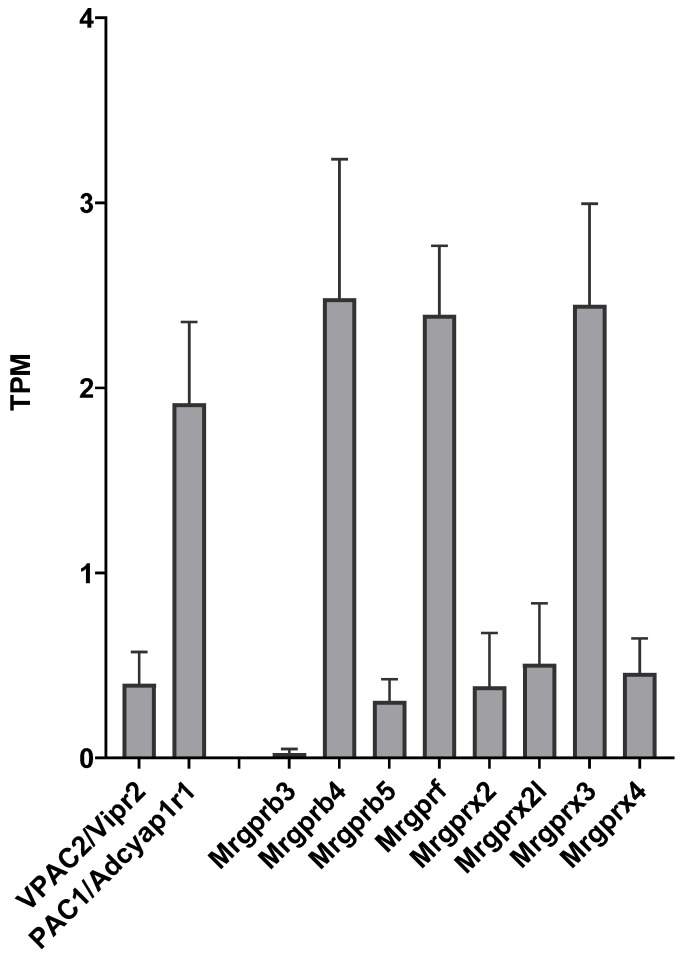
Transcripts Per Million (TPM) values for receptors with known or potential affinity for PACAP in untreated control trigeminal ganglion (TG) cell cultures. Data represent mean ± SD (*n* = 3).

Transcripts of the known receptors of PACAP, PAC1 (Adcyap1r1) and VPAC2 receptors, as well as several Mrgpr receptors were detected in most samples as presented in Figure 1. These receptors were not differentially expressed in the PACAP-38- and PACAP(6-38)-treated groups compared to the control. VPAC1 was not expressed in either group. 

### 2.2. Differentially-Expressed (DE) Genes in Both PACAP-38- and PACAP(6-38)-Treated Trigeminal Ganglion Cultures

**Figure 2:**
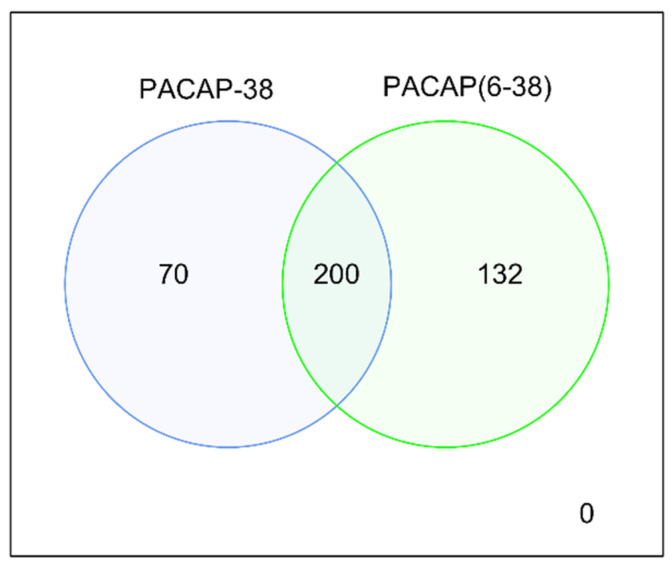
Numerical representation of differentially expressed genes for PACAP-38 and PACAP6-38. The expression of genes was compared to the respective untreated control groups.

Figure 2 shows the numerical analysis of differentially expressed genes. Sample collection 6 h after the treatment yielded 200 common differentially expressed genes for PACAP-38 and PACAP6-38. For PACAP-38, 70 other DE genes, for PACAP6-38 132 other DE genes were found at the 6 h samplings. All DE genes are listed in Table A1.

**Figure 3:**
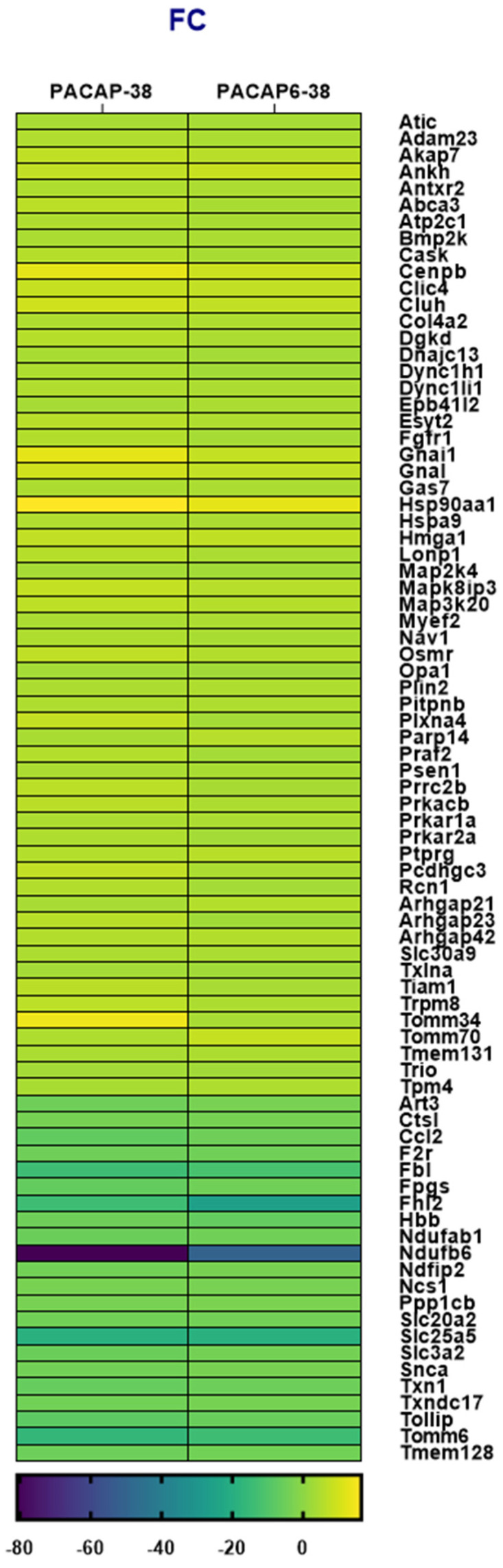
Heatmap of fold change (FC) values for differentially expressed (DE) genes shared in trigeminal ganglion (TG) cell cultures treated with PACAP-38 or PACAP(6-38) compared to the control group (1 µM, 6 h). *p* < 0.05 was considered as significantly different. (See the list of gene name abbreviations in Appendix A). DE genes associated with neurological disorders were selected from total hit according to databases (https://rgd.mcw.edu/; https://www.genecards.org/; https://www.ncbi.nlm.nih.gov last accessed date: 10 April 2021).

Figure 3 shows common DE genes potentially involved in neurological pathophysiology for PACAP-38 and PACAP(6-38) treatments compared to the untreated control cell culture. Noteworthy findings in the DE list are Cenpb, Gnal, Hsp90aa1, Hmga1, Tomm70, Gnai1 and Tomm34 which were upregulated regarding FC value in the case of both PACAP-38- and PACAP(6-38)-treated TG cell culture (Figure 2). It is highly notable in both neuropeptide-treated cell cultures that Ndufb6 (NADH:ubiquinone oxidoreductase subunit B6) was extensively downregulated compared to the control group (value of FC was −50.7 and −80.9 for PACAP-38- and PACAP(6-38)-treated cells, respectively) and Trpm8 was upregulated in both cases. Fbl (Fibrillarin); Fhl2 (four and a half LIM domains 2), Slc25a5 (solute carrier family 25 member 5), Tomm6 (translocase of outer mitochondrial membrane 6) were also highly downregulated in both. Listed abbreviations can be found in Appendix A.

### 2.3. Pathway Analysis with Common DE Genes

**Figure 4:**
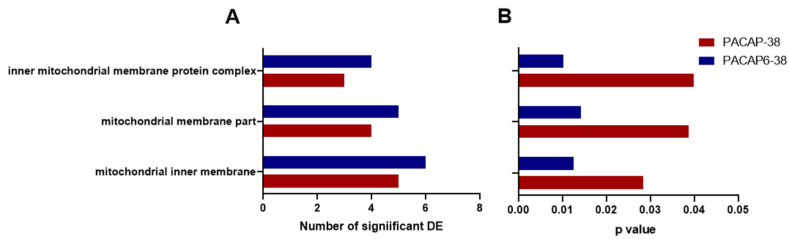
Highlighted GO analysis results which were significant in both PACAP-38- (red) and PACAP(6-38)- (blue) treated trigeminal ganglion cells (1 µM, 6 h). Panel **A** represents the number of differentially expressed (DE) genes related to each GO term, while panel **B** shows *p* values for the identical terms (*p* < 0.05).

Figure 4 shows the results for GO analyses of downregulated genes. This search highlighted mitochondrial dysfunction, regarding *p*-values for each term or number of significant DE genes for both administration types (Figure 4A,B).

**Figure 5:**
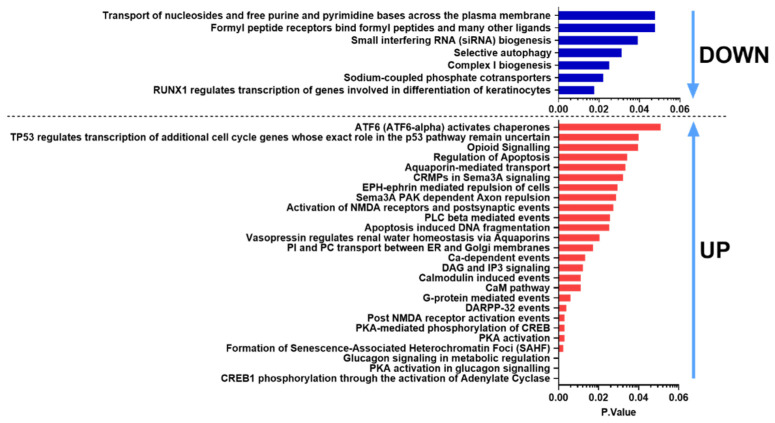
Significantly affected Reactome pathways found for both PACAP-38 and PACAP(6-38)-treated cultures ranked according to the *p*-value. Blue bars indicate downregulated, while red bars show upregulated pathways.

Reactome analysis was used to determine the intracellular pathways the DE genes were involved in. Figure 5 shows pathways for the common DE genes gained from the Reactome database. CREB1 phosphorylation through the activation of adenylate cyclase, protein kinase A (PKA) activation in glucagon signaling, glucagon signaling in metabolic regulation, PKA activation, PKA-mediated phosphorylation of CREB, Post NMDA receptor activation events were found in the case of upregulated genes. Ca-dependent events were upregulated, while Complex I biogenesis was downregulated in response to both PACAP-38- and PACAP(6-38)-treatment, referring to potential mitochondrial dysfunction mechanism. The results of GO (Figure 4) and Reactome analysis also revealed inhibiting effects of both peptides on mitochondrial functions supporting this concept. 

**Figure 6:**
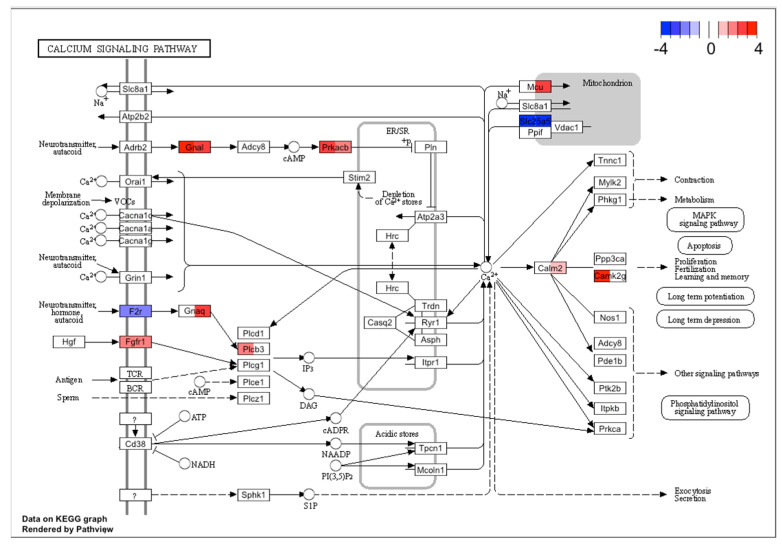
The KEGG Ca^2+^ signaling pathway was significantly altered in both PACAP-38 and PACAP(6-38)-treated trigeminal ganglion cells. Rectangles in color indicate genes that were significantly changed after treatment (left sides of rectangles represent PACAP-38, while right sides stand for PACAP(6-38)). The red and blue color scale show normalized fold change values for each differentially expressed gene. The KEGG Ca^2+^ signaling pathway also significantly implicated by ranked list enrichment performed on all genes can be found in Supplementary Figure S1.

In the KEGG analysis, one of the common pathway results was the calcium signaling pathway. Figure 6 highlights the common DE genes in this signaling pathway. In both cases GnaI, Prkacb were upregulated, and F2R, Slc25a5 were downregulated. This result also highlighted the possible negative effect on the mitochondria. For TG culture treated with PACAP-38, Figure 6 showed Plcb3 as upregulated, in contrast to TG culture administered with PACAP(6-38), while Gnaq was significantly upregulated in the PACAP(6-38) but not PACAP-38 group. Expression of some genes also presented with a distinct pattern: Calm2, MCU were upregulated in the PACAP(6-38) and Camk2g in the PACAP-38 treated cells. These results showed the effect of PACAP-38/PACAP(6-38) on the calcium signaling pathway. The KEGG Ca^2+^ pathway was also implicated significantly when ranked list enrichment was performed, which takes into account not only DE genes but all genes whose transcripts were detected. The Ca^2+^ pathway containing all genes can be found in Supplementary Figure S1.

## 3. Discussion

This is the first description of transcriptomic changes of rat trigeminal ganglion cells in response to PACAP-38 related to mitochondrial dysfunction and neuroinflammatory mechanisms potentially mediated by calcium signaling. Moreover, these alterations were similar after treatment with PACAP(6-38), known to be PAC1 receptor antagonist at the cloned receptor, which indicates that trigeminal ganglion cell activation is independent of the PAC1 receptor. In our previous study, both PACAP-38 and PACAP(6-38) were found to induce increased intracellular Ca^2+^ levels in the same trigeminal ganglion cell culture [36]. Interestingly, similar agonistic effects for the two peptides were found on sensory nerve terminals of the rat trachea and human cytotrophoblast cells [35,37], mouse macrophages [38], chicken chondroblasts [39] and rat mast cells [7]. The stimulating effect of PACAP(6-38) was a surprising finding, since it is a well-established antagonist on the identified and cloned receptors of PACAP-38, PAC1 and VPAC1/2 receptors, heterologously expressed in CHO, Cos7 cells and *Xenopus oocytes* [7,36,40]. Our previous and present results raises the possibility that PACAP-induced trigeminovascular activation involved in migraine [21] is not mediated by these known receptors. This virtually contradictory, stimulating effect of PACAP(6-38) might be explained by an action on a receptor other than PAC1 [22]. The MrgB3 receptor was proposed as a potential target of both peptides to induce rat meningeal mast cell activation [7], but the involvement of a different PAC1 splice variant with modified binding site cannot be excluded.

Among the known receptors of PACAP-38, transcripts of PAC1 and VPAC2 receptors were detected in trigeminal ganglion cell cultures which is in line with previously reported data [27]. We have also detected several subtypes of the Mrgpr family which could be putative targets mediating the shared effects of PACAP-38 and PACAP(6-38). Mrgprs constitute a large, diverse family of G-protein coupled receptors originally described on primary sensory neurons [8], but expressed by other cell types including mast cells [6,7,41]. The endogenous ligands of these receptors are still not well defined, but various Mrgprs can be activated by amino acids or peptides, e.g., MrgprD by β-alanine or MrgprX1 by proenkephalin and proopiomelanocortin cleavage products [34]. In sensory neurons Mrgprs have been implicated in the transduction of pain and itch, as well [42]. However, interestingly, a small subset of MrgprB4-positive C-fibers were also identified as transmitting gentle stroking, but not noxious mechanical stimuli [43]. In mast cells, Mrgprs are responsible for the non-IgE-mediated activation by basic secretagogues [41]. In particular, degranulation induced by PACAP, PACAP(6-27) or PACAP(6-38) was shown to be mediated by MrgprX2 in human and MrgprB3 receptors in rat mast cells [6,7]. In our TG culture, we have detected the presence of MrgprB3 and transcripts of seven other Mrgprs. According to our results, we cannot exclude totally the role of PAC1 receptor, because we did not have a group for inhibiting PAC1 receptor or deleting PAC1 receptor in cells. It can be suggested, that either MrgprB3 or one of the other Mrgpr subtypes were activated by PACAP-38 and PACAP(6-38), but further experiments are needed to confirm this mechanism. 

In order to identify the PACAP-induced intracellular signaling pathways in primary sensory neurons functional enrichment analysis of commonly altered DE genes by PACAP-38 and PACAP(6-38) pointed to significantly altered calcium signaling and PKA activation. Since PAC1 receptors can activate adenylate cyclase and phospholipase C signaling pathways as well [4], these data does not conclusively exclude the role of PAC1 receptors in the response to PACAP. However, as mentioned above, it is plausible that a common target is mediating the effects of the two peptides. Both calcium and PKA of these signaling pathways can be associated with increased nociceptor sensitivity [44,45] which supports the pronociceptive effects of PACAP. PKA activation can increase the sensitivity of transient receptor potential vanilloid 1 (TRPV1) channels, a key transducer of noxious heat and chemical stimuli [46,47] and voltage-gated sodium channels [48,49], which result in lowered activations thresholds and increased suprathreshold responses of nociceptive neurons. Intracellular calcium binding to calmodulin can activate calmodulin-dependent kinase (CaMKII) which was shown to be involved in changes of synaptic plasticity behind chronic pain conditions [45]. Increased intracellular calcium levels can also affect mitochondrial function. Functional enrichment analysis results point to mitochondrial alteration associated with a mitochondrial electron transport chain dysfunction. In particular, the B6 subunit of NADH: ubiquinone oxidoreductase (Complex I) was strongly downregulated by both treatments. This is an intriguing finding which can also link the effect of PACAP to migraine as metabolic changes and mitochondrial dysfunction such as decreased activity of Complex I, III, IV and citrate synthase have been detected in migraine patients [50,51]. Our recent study investigating the transcriptome of peripheral blood mononuclear cells of migraine patients also revealed that the mitochondrial electron transport chain was significantly affected even in headache-free periods and during headache when compared to healthy control samples [52]. It is plausible therefore that the migraine headache-inducing effect of PACAP is generated with the involvement of mitochondria. Regarding the importance of mitochondria in nociceptive sensory neurons, there is accumulating evidence demonstrating mitochondrial dysfunction in several chronic pain conditions and pain is a common complaint in patients with mitochondrial diseases [53,54,55]. Mitochondria together with the endoplasmic reticulum are key regulators of intracellular calcium homeostasis which set neuronal excitability. Moreover, mitochondrial dysfunction can lead to increased generation of reactive oxygen species which can also contribute to nociceptor sensitization by acting on multiple targets [56]. There is direct evidence that experimental inhibition of mitochondrial complex III in airway C fibers resulted in increased excitability by activation of TRP channels and protein kinase C [57,58]. Moreover, reactive oxygen species and the release of mtDNA can induce an inflammatory reaction promoting the sensitization of nociceptors. 

Other interesting pain-related genes in the DE list include upregulation of the TRPM8 ion channel, which is a menthol- and cold-sensitive ion channel expressed in both dorsal root and trigeminal ganglion cells [59,60]. TRPM8 is expressed by both nociceptive and non-nociceptive sensory neurons, co-expressed with the TRPV1 ion channels in the first case [61,62,63]. Under physiological conditions TRPM8 is responsible for detecting innocuous and noxious cold temperatures [64,65,66], but it can also reduce the activation of nociceptors by other stimuli, which explains the alleviation of pain by cooling or menthol. However, there are also data showing the involvement of TRPM8 in cold allodynia in chronic inflammatory and neuropathic pain models [67]. Remarkably, there is also a possible role of TRPM8 in migraine as several large genome-wide association studies identified single nucleotide polymorphisms within and near the TRPM8 gene which confer a reduced risk of migraine [68,69,70]. Cutaneous application of menthol reduced the headache in migraine patients [71]. Likewise, another TRPM8 receptor agonist icilin reversed allodynia in an animal model of dural sensitization [72]. The relevance of the upregulation of Trpm8 after PACAP-38- or PACAP(6-38)-treatment is not yet clear as there are no literature data linking TRPM8 ion channel function with PACAP. There is, however, an interesting potential connection between TRPM8 channels and mitochondrial dysfunction. The presence of TRPM8 was shown in the endoplasmic reticulum (ER) of several cell types, including human keratinocytes [73], bronchial epithelial cells [74], prostate cancer cells [75] as well as mouse vascular smooth muscle cells [76]. In these studies, TRPM8 channels in the ER were shown to participate in the regulation of calcium homeostasis between intracellular compartments and influence mitochondrial function. While the presence of TRPM8 in the ER has not yet been reported in sensory neurons, other TRP channels, such as TRPV1 are expressed in both the plasma and ER membranes of dorsal root ganglion cells [77,78] therefore it is plausible that the ER TRPM8 can affect mitochondrial Ca^2+^ levels in trigeminal neurons, as well. 

**Figure 7:**
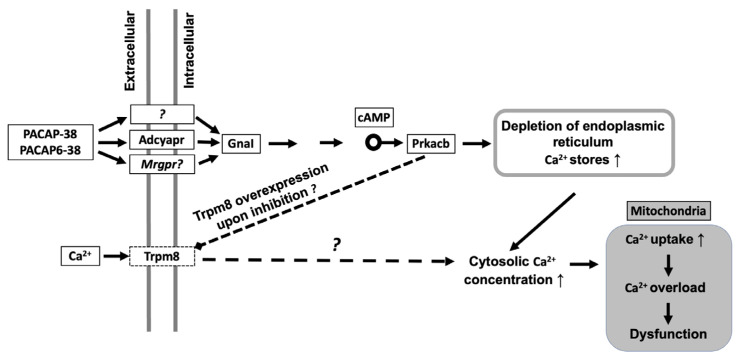
Schematic representation of the hypothetical mechanisms initiated by PACAP in trigeminal ganglion cells. PACAP-38/PACAP6-38 activate G- protein coupled receptors (GPCR). Activation of Guanine nucleotide-binding protein G(i) subunit alpha-1 (GnaI) leads to protein kinase A (PKA; Protein Kinase cAMP-Activated Catalytic Subunit Beta—Prkacb) activation via increased cAMP levels. Phosphorylation by PKA may induce TRPM8 channel inhibition which could cause overexpression of TRPM8 ion channel. Calcium overload could lead to suppressed Complex I biogenesis.

Figure 7 summarizes our findings in a schematic graph. Both neuropeptides act on G-protein coupled receptors (GPCR) which could either be a different target structure such as Mrgpr receptors or a splice variant of PAC1/VPAC2 receptors. GPCR trigger Guanine nucleotide-binding protein G(i) subunit alpha-1 (GnaI). GnaI activates protein kinase cAMP-Activated Catalytic Subunit Beta via increased cAMP levels. Phosphorylation by PKA may induce TRPM8 channel inhibition [79] and this inhibitory activity might potentiallly cause overexpression of TRPM8, potentiating calcium overload in mitochondria resulting Complex I suppression.

In conclusion, transcriptomic changes induced by PACAP-38 and PACAP6-38 in cultured TG cells indicated cellular processes which can be associated with mechanisms occurring in migraine patients, in particular nociceptive sensitization and mitochondrial dysfunction. A limitation of our approach is the lack of functional confirmation of the affected cellular pathways which needs future research. Results, reported in this article are supported by previous human and animal experimental data on the mechanisms of migraine headache. Although, we could not identify the target of PACAP-38 for sensory neuronal activation, the common transcriptome alterations with PACAP(6-38) can potentially suggest a PAC1 independent shared pathway. These results open novel perspectives for antimigraine drug research connected to mitochondrial function. 

## 4. Materials and Methods

### 4.1. Primary Cultures of TG Neurons

Primary cell cultures of TG neurons were made from 1–4-day-old Wistar rat pups as described elsewhere [80]. TG cells were dissected in ice-cold phosphate-buffered solution (PBS), incubated for 35 min at 37 °C in PBS containing collagenase (Type XI, 1 mg/mL) and then in PBS with deoxyribonuclease I (1000 units/mL) for 8 min. The ganglia were washed with Ca^2+^ and Mg^2+^-free PBS and dissociated by trituration. TG cells were plated on poly-D-lysin-coated glass coverslips and grown in a nutrient-supplemented medium for the experiment. The cell culture medium contained 180 mL Dulbecco’s- Modified Eagle Medium (D-MEM), 20 mL horse serum, 20 mL fetal bovine albumin, 2 mL insulin-transferrinselenium- S, 3.2 mL putrescin dihydrochloride (100 µg/mL), 20 µL triiodo-thyronine (0.2 mg/mL), 1.24 mL progesterone (0.5 mg/mL), 100 µL penicillin, 100 µL streptomycin and nerve growth factor (NGF, 200 ng/mL). The coverslips were maintained at 37 °C in an atmosphere containing 5% CO_2_. The cell cultures were incubated with 1 µM PACAP-38 or PACAP6-38. Untreated cultures served as controls. After 6 h after PACAP-38 or PACAP6-38 administration, samples were collected for RNA isolation. Conditions were repeated in triplicates. 

### 4.2. RNA Isolation and Quality Control

Total RNA isolation and purification was performed as previously described [81] applying the phenol-based TRI Reagent procedure (Molecular Research Center, Cincinnati, OH, USA), up to the step of acquiring the RNA containing aqueous layer. The aqueous phase was mixed with an equal volume of absolute ethanol and was loaded into Zymo-Spin™ IICR Column. Direct-zol RNA MiniPrep kit (Zymo Research, Irvine, CA, USA) was used according to the manufacturer’s protocol including the optional on-column DNase digestion. Qubit 3.0 (Invitrogen, Carlsbad, CA, USA) was used for RNA concentration measurement. The RNA quality verification was carried out with TapeStation 4200 using RNA ScreenTape (Agilent Technologies, Santa Clara, CA, USA). Sequencing libraries were prepared from high quality (RIN > 8) RNA samples.

### 4.3. Illumina Library Preparation and Sequencing

The library for Illumina sequencing was prepared using QuantSeq 3′ mRNA-Seq Library Prep Kit FWD for Illumina (Lexogen, Vienna, Austria). 400 ng of total RNA was used as input for first strand cDNA generation using oligodT primer followed by RNA removing. Thereafter, the second strand synthesis is initiated by random priming and the products were purified with magnetic beads. Finally, the libraries were amplified and barcoded using PCR. All libraries were assessed on the TapeStation 4200 (Agilent Technologies, Santa Clara, CA, USA) to examine if adapter dimers formed during PCR. The QuantSeq libraries were sequenced using the Illumina NextSeq550 platform to produce 75 bp single end reads. 

### 4.4. Bioinformatics

The sequencing reads were aligned against the *Rattus norvegicus* reference genome (Rnor 6.0 Ensembl release) with STAR v2.5.3a [82]. After alignment, the reads were associated with known protein-coding genes and the number of reads aligned within each gene was counted using HTSeq library v0.11.1 [83]. Gene count data were normalized using the trimmed mean of M values (TMM) normalization method of the edgeR R/Bioconductor package (v3.28, R v3.6.0, Bioconductor v3.9) [84]. Data were further log transformed using the voom approach for statistical evaluation [85] in the limma package [86]. Fold change (FC) values between the compared groups resulting from linear modeling process and modified *t*-test *p*-values were produced by the limma package. When determining differentially expressed (DE) genes, filtering thresholds were set to FC 2 and *p*-value 0.05 when the PACAP-38 treatment was compared to the untreated control group, and to FC 1.5 and *p*-value 0.001 for the PACAP(6-38) versus untreated control comparison (*p*-values are provided after correction for multiple comparisons by the Benjamini-Hochberg method. Normalized counts were represented as transcripts per million (TPM) values. Functional analysis (annotations of genes) was performed using the Gene Ontology (GO), Kyoto Encyclopedia of Genes and Genomes (KEGG), and Reactome databases. Detection of functional enrichment was performed in the differentially expressed gene list (DE list enrichment: Fisher’s exact test for GO, hypergeometric test for KEGG and Reactome) and towards the top of the list when all genes have been ranked according to the evidence for being differentially expressed (ranked list enrichment: non-parametric Kolmogorov-Smirnov test for GO and KEGG, hypergeometric test for Reactome) applying the topGO v2.37.0, ReactomePA v1.30.0, gage v2.36.0 packages. The pathview package v1.26.0 [86] was used to visualize mapping data to KEGG pathways. All data for each gene at different time can be found in Supplementary section. 

## Data Availability

All RNA-Seq data sets generated as part of this study will be publicly available at the European Nucleotide Archive (https://www.ebi.ac.uk/ena, accessed on 1 Ocotber 2021), under accession number PRJEB47291.

## Supplementary Materials

The following are available online at https://www.mdpi.com/article/10.3390/ijms23042120/s1.

## Appendix A

**Table A1:** List of DE Gene Names.

**Abbreviation**	**Description**
Abca3	ATP binding cassette subfamily A member 3
Adam23	ADAM metallopeptidase domain 23
Akap7	A-kinase anchoring protein 7
Ankh	ANKH inorganic pyrophosphate transport regulator
Antxr2	ANTXR cell adhesion molecule 2
Arhgap21	Rho GTPase activating protein 21
Arhgap23	Rho GTPase activating protein 23
Arhgap42	Rho GTPase activating protein 42
Art3	ADP-ribosyltransferase 3
Atic	5-aminoimidazole-4-carboxamide ribonucleotide formyltransferase/IMP cyclohydrolase
Atp2c1	ATPase secretory pathway Ca^2+^ transporting 1
Bmp2k	BMP-2 inducible kinase
Cask	calcium/calmodulin dependent serine protein kinase
Ccl2	C-C motif chemokine ligand 2
Cenpb	centromere protein B
Clic4	chloride intracellular channel 4
Cluh	clustered mitochondria homolog
Col4a2	collagen type IV alpha 2 chain
Ctsl	cathepsin L
Dgkd	diacylglycerol kinase, delta
Dnajc13	DnaJ heat shock protein family (Hsp40) member C13
Dync1h1	dynein cytoplasmic 1 heavy chain 1
Dync1li1	dynein cytoplasmic 1 light intermediate chain 1
Epb41l2	erythrocyte membrane protein band 4.1-like 2
Esyt2	extended synaptotagmin 2
F2r	coagulation factor II (thrombin) receptor
Fbl	fibrillarin
Fgfr1	Fibroblast growth factor receptor 1
Fhl2	four and a half LIM domains 2
Fpgs	folylpolyglutamate synthase
Gas7	growth arrest specific 7
Gnai1	G protein subunit alpha i1
Gnal	G protein subunit alpha L
Hbb	hemoglobin subunit beta
Hmga1	high mobility group AT-hook 1
Hsp90aa1	heat shock protein 90 alpha family class A member 1
Hspa9	heat shock protein family A (Hsp70) member 9
Lonp1	lon peptidase 1, mitochondrial
Map2k4	mitogen activated protein kinase kinase 4
Map3k20	mitogen-activated protein kinase kinase kinase 20
Mapk8ip3	mitogen-activated protein kinase 8 interacting protein 3
Myef2	myelin expression factor 2
Nav1	neuron navigator 1
Ncs1	neuronal calcium sensor 1
Ndfip2	Nedd4 family interacting protein 2
Ndufab1	NADH:ubiquinone oxidoreductase subunit AB1
Ndufb6	NADH:ubiquinone oxidoreductase subunit B6
Opa1	OPA1, mitochondrial dynamin-like GTPase
Osmr	oncostatin M receptor
Parp14	poly (ADP-ribose) polymerase family, member 14
Pcdhgc3	protocadherin gamma subfamily C, 3
Pitpnb	phosphatidylinositol transfer protein, beta
Plin2	perilipin 2
Plxna4	plexin A4
Ppp1cb	protein phosphatase 1 catalytic subunit beta
Praf2	PRA1 domain family, member 2
Prkacb	protein kinase cAMP-activated catalytic subunit beta
Prkar1a	protein kinase cAMP-dependent type I regulatory subunit alpha
Prkar2a	protein kinase cAMP-dependent type II regulatory subunit alpha
Prrc2b	proline-rich coiled-coil 2B
Psen1	presenilin 1
Ptprg	protein tyrosine phosphatase, receptor type, G
Rcn1	reticulocalbin 1
Slc20a2	solute carrier family 20 member 2
Slc25a5	solute carrier family 25 member 5
Slc30a9	solute carrier family 30 member 9
Slc3a2	solute carrier family 3 member 2
Snca	synuclein alpha
Tiam1	TIAM Rac1 associated GEF 1
Tmem128	transmembrane protein 128
Tmem131	transmembrane protein 131
Tollip	toll interacting protein
Tomm34	translocase of outer mitochondrial membrane 34
Tomm6	translocase of outer mitochondrial membrane 6
Tomm70	translocase of outer mitochondrial membrane 70
Tpm4	tropomyosin 4
Trio	trio Rho guanine nucleotide exchange factor
Trpm8	transient receptor potential cation channel, subfamily M, member 8
Txlna	taxilin alpha
Txn1	thioredoxin 1
Txndc17	thioredoxin domain containing 17
