# Supplementary figures and images for: PACAP-38 Induces Transcriptomic Changes in Rat Trigeminal Ganglion Cells Related to Neuroinflammation and Altered Mitochondrial Function Presumably via PAC1/VPAC2 Receptor-Independent Mechanism
Source: Int J Mol Sci. 2022 Feb 14;23(4):2120. doi: 10.3390/ijms23042120 (PMC8874739; doi:10.3390/ijms23042120)

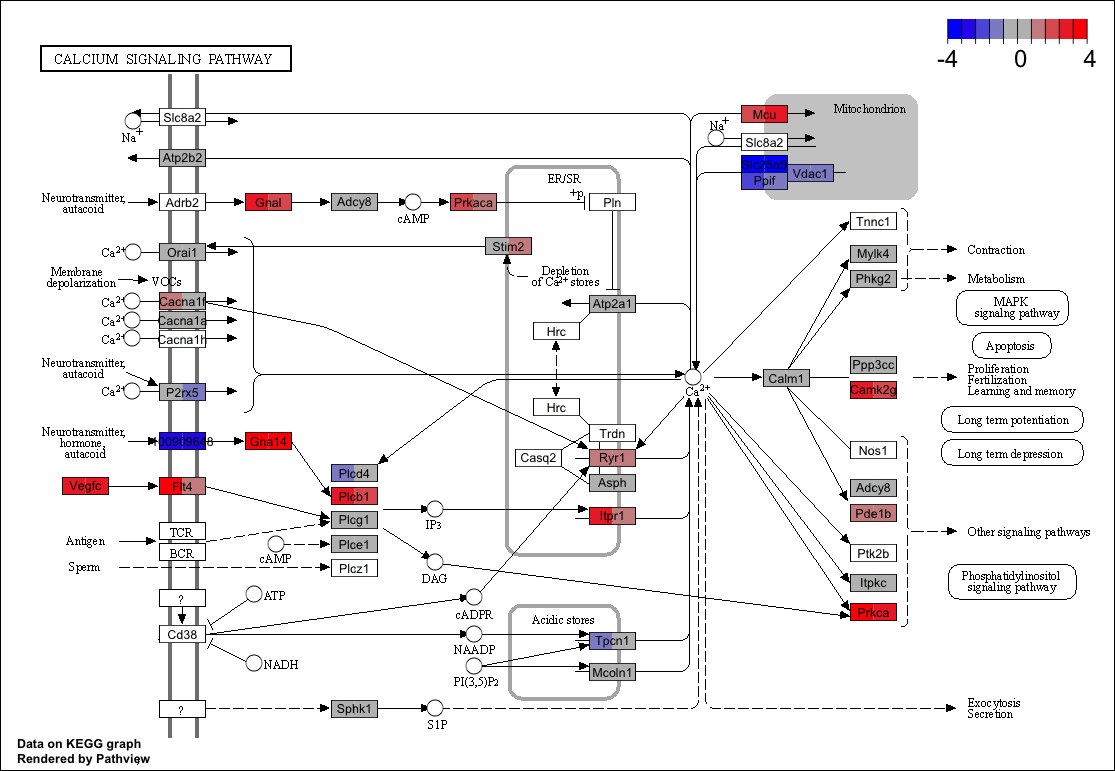

Supplement: Supplementary file 1 [file ijms-23-02120-s001.zip › Figure S1AllDEgenesinCalciumSignalingpathway.png]
